# Rotating panoramic view: interaction between visual and olfactory cues in ants

**DOI:** 10.1098/rsos.150426

**Published:** 2016-01-27

**Authors:** Mai Minoura, Kohei Sonoda, Tomoko Sakiyama, Yukio-Pegio Gunji

**Affiliations:** 1 School of Fundamental Science and Engineering, Waseda University, Tokyo, Japan; 2 Research Organization of Science and Technology, Ritsumeikan University, Shiga, Japan

**Keywords:** foraging, ant, landmark

## Abstract

Insects use a navigational toolkit consisting of multiple strategies such as path integration, view-dependent recognition methods and olfactory cues. The question arises as to how directional cues afforded by a visual panorama combine with olfactory cues from a pheromone trail to guide ants towards their nest. We positioned a garden ant *Lasius niger* on a rotating table, whereon a segment of a pheromone trail relative to the stationary panorama was rotated while the ant walked along the trail towards its nest. The rotational speed of the table (3 r.p.m.) was set so that the table would rotate through about 90° by the time that an ant had walked from the start to the centre of the table. The ant completed a U-turn at about this point and so travelled in a nest-ward direction without leaving the trail. These results suggest that the ants persist on the pheromone trail and use visual input to determine their direction of travel along the trail.

## 1. Introduction

Insects use many information sources to forage for food, learn the location of places, revisit places and return home to a nest [1–3]. Ants and bees are known to use information from the visual panorama for navigation [4–6]. For example, bees can extract location information from an array of visual objects [7] as well as remember the colour of objects [8] and their texture [9]. Ants use odour information sources, such as trail pheromones [10] and odour plumes emanating from the nest [11]. Desert ants can monitor their positions relative to a nest or to a previously visited food source using a form of reckoning known as path integration [12,13].

These types of navigational information are integrated to increase the efficiency of the goal-seeking procedure [14]. In the ant *Lasius niger*, route memory and trail pheromones act synergistically during foraging to increase walking speed and straightness as well as to maintain trail pheromone deposition [15]. *Cataglyphis fortis*, the desert ant, uses path integration for long-distance navigation and relies on visual and odour information to discern a nest entrance [11]. The ant also calibrates a systematic search pattern using path integration information when it is deprived of additional external cues [16]. Other desert ants also use both vector-based and visually guided navigation [17].

Ants are known to rely on a navigational toolkit consisting of multiple strategies [18,19]. By employing information sources for multiple uses, how do ants adapt their navigation strategy where different kinds of cues are afforded, and can the strategies conflict? Grüter *et al*. [20] showed that route memory overrides trail pheromones after only one visit to a food source when the two cues are inconsistent. Recent works have focused on the interaction between path integration and the visual panorama and have shown that when they are put into conflict, ants integrate these strategies together rather than switching either one to ‘using’ or ‘off’ [21–23].

Here, we investigate the interaction between the visual panorama and odour trails. The ants' path between nest and feeder crossed a circular table on which a straight pheromone trail had been laid. Once an ant left the feeder and started to follow the trail, the table was made to rotate so that visual cues indicating the direction of the nest came into increasing conflict with the orientation of the trail. Ants tended to stick to the pheromone trail despite the rotation and they corrected their direction by making U-turns on the trail. We used several variants of this methodology to investigate how the ant is guided by these two cues on its trip back to the nest. Therefore, the rotating panorama construction was useful for precisely estimating an ant's ability to adapt to the complexity of its environment where different cues guide the ant in different directions.

## 2. Material and methods

### 2.1 Study species and experimental apparatus

**Figure 1.**
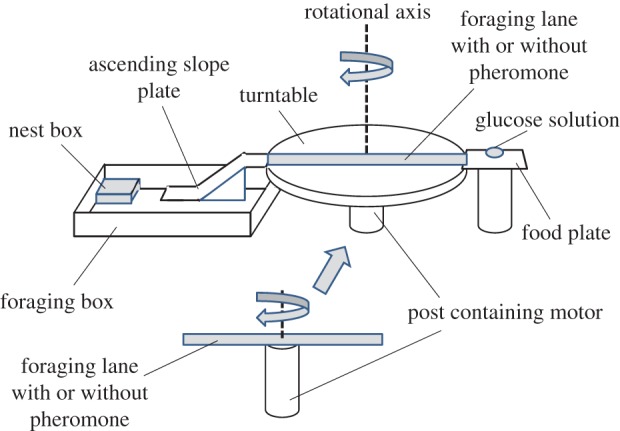
The apparatus based on the rotating table experiment. The number of ants in each experiment was 30. The diagram shows the experimental apparatus. In each experimental set-up, only the rotational structure was replaced. The table was rotated at 3 r.p.m. (EXP 1-1) or was not rotated (EXP 1-2). In EXP 2, the table was rotated very slowly at 1 r.p.m. In EXP 3, the turntable was covered by a white paper wall. In EXP 4-1 and 4-2, the turntable was replaced with a foraging lane. Each detailed set-up is shown in figures 2*a*, 4*a*–8*a*.

**Figure 2.**
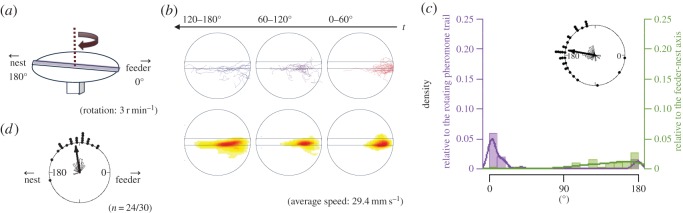
The apparatus and results from EXP 1-1. (*a*) The rotational structure in the experimental apparatus of EXP 1-1. (*b*) Each trajectory (top) and the density distribution (bottom) of ants' walking on the turntable. The trajectories are shown on the disc as though the disc had not rotated. Each trajectory is divided into four portions and the first three are shown. First portion, the trajectory taken during rotation from 0° to 60° (top or bottom right); second portion, from 60° to 120° (top or bottom centre); third portion, from 120° to 180° (top or bottom left) and fourth portion, over 180° (omitted). The density distribution of the trajectory is graded from more dense (red) to less dense (yellow to white). The average walking speed of all ants is calculated and shown at the bottom right. (*c*) The histogram of the angles formed by the point where the ants reached the edge of the table or lane, relative to the feeder–nest axis (green) and relative to the rotating pheromone lane (violet). For the former one, the 0° is set to the start point (i.e. feeder side), and for the latter, the 0° is set to the lane direction (refer to figure 3*a*,*b*). The distribution of the angle relative to the feeder–nest axis is also represented by the circular graph. An arrow represents the mean endpoint angle, the resultant arrow length corresponds to the angle concentration, and the dots on the exterior of the circle indicate individual data points. In EXP 1-1, the peak of the histogram relative to the feeder–nest axis is around 180° and that relative to the rotating pheromone lane is around 0°. This indicates that most ants completed a U-turn (returned at 180° relative to the feeder–nest axis) and returned to the starting point (0° relative to the pheromone lane). (*d*) The angle formed by the lane when the ants U-turn relative to the direction of the line between the feeder and the nest. The number of U-turns over the total number of ants is shown at the bottom right. We defined U-turn as the trajectory that meets the following three conditions: (i) the angle relative to the rotating pheromone lane is less than 15°, which means the ant came back around to the starting side; (ii) ants stepped forward to the nest side more than 30 mm; and (iii) ants made just one turn.

We studied three *L. niger* colonies collected on the Kobe University campus. The colonies were queenless and included 900–1200 workers. The colonies were maintained at room temperature (25°*C*±1°*C*) and were starved for 5–6 days prior to the experiment. The food provided in the experiment was a glucose solution (50% w/w), which was presented on a feeder (2×8 cm and 1 cm high). The foraging box (35×25 cm and 4 cm high) was connected to the feed plate with a plastic slope plate and a foraging lane (pheromone-coated or uncoated; rotated or not rotated; 30×3 cm) on a turntable (30 cm diameter) or a turn bridge (30×3 cm) (figures 1, 2*a*, 4*a*, 5*a*, 7*a* and 8*a*). In the main experiment, the turntable was rotated in a stationary room giving the ants a changing view of the visual panorama. Before the experiment began, the colony had no opportunity to learn the location of the nest relative to room cues.

**Figure 3.**
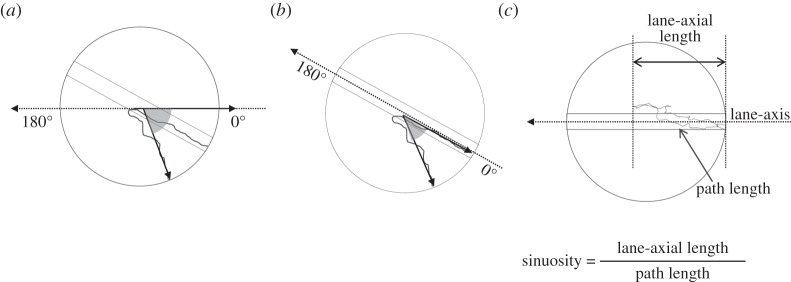
Schematic diagram for the definition of angle relative to the feeder–nest axis and the pheromone lane and sinuosity. (*a*) The angle relative to the feeder–nest axis, used for figures 2, 4–8. The basing points are set on the feeder side as 0° and on the nest side as 180°. (*b*) The angle relative to the rotating pheromone lane, used for figures 2, 4–8. The basing points are set on an ant's start side of the lane as 0° and on the other side of the lane as 180°. (*c*) The lane-axis length and the travel length used for calculating sinuosity.

To obtain a pheromone sheet, a foraging box was connected to a lane covered with an acid-free paper. The colony was allowed to explore the lane and to access the turntable on a platform at the end of the lane for 20 min. During this period, at least 15 ants contributed to a pheromone trail which lasted about 1 h. To ensure ants encountered a good trail, a new pheromone sheet was used for each 30 min.

The turntable was surrounded by a white paper under one experimental condition (figures 1 and 6*a*). The turn stages (turntable or turn bridge) were rotated using a synchronous motor (type D-5N, Nidec Servo Corporation, Tokyo). Because the direction of turntable rotation did not significantly affect the experiment, we used a clockwise rotation. The experiments were performed in a room with artificial lighting. The room included different types of laboratory equipment and furniture.

The experiments were recorded with a video camera (GZ-MG740, Victor). The camera was placed horizontally and 80 cm above the centre of the turntable (EXP 1, 2) or turn bridge (EXP 4) and 120 cm above the centre of the turntable in a hole on the upper portion of the white column (EXP 3). The camera was pointing downwards to record a plan view of the rotating disc. The angle data were acquired with Avidemux2 and the GIMP toolbox.

For the experiments, the ants were used one at a time for each trial. After a trial, the ants were eliminated; hence, the ants were naive to the turntable and to the visual panorama seen from the turntable in all experiments. That is, the colony had no opportunity to learn the location of the nest relative to the room cues before the experiments began. Some ants, however, could have experienced the visual panorama seen from the non-rotated lane connecting the feeder and the foraging box because they were employed in the preparation of the pheromone-coated lane. In principle, all ants could learn the panorama during the outward trip.

All experiments were set up as follows (figure 1). A turn stage and an ascending slope were connected with a foraging lane on a turntable (EXP 1-1, 1-2, 2 and 3) or a bridge (EXP 4-1 and 4-2). The foraging lane led to the feeder. The turntable was covered with a sheet of acid-free paper, which was frequently replaced with a new sheet. The foraging lane was deposited with trail pheromone in some experiments but not in others. Soon after an ant entered the foraging lane, the ascending slope was detached from the turntable to prevent other ants from entering the lane. During the outbound journey, the turntable was not rotated. Thus, an ant could reach the glucose solution on the food plate by going straight along the foraging lane. The turntable began rotating when the ant re-entered the foraging lane after feeding. The trial was considered complete when the ant reached the edge of the turntable (or foraging lane).

#### 2.1.1 Experiment 1: rotating table

**Figure 4.**
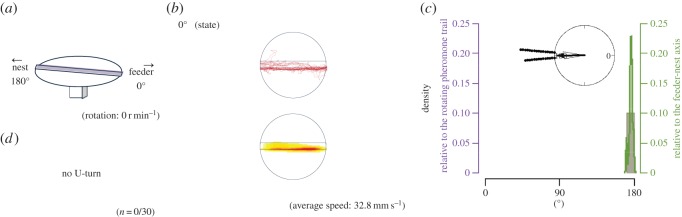
The apparatus and results from EXP 1-2. Explanations of diagrams and graphs (*a*–*d*) are the same as in figure 2, except different explanations are added as follows: (*a*) the rotational structure in the experimental apparatus of EXP 1-2. (*b*) The turntable was not rotated, thus the trajectory pattern was not divided. (*c*) In EXP 1-2, the two peaks overlapped with each other because all ants went straight to the nest side.

We investigated how ants adopt their foraging behaviour by using a rotating panorama. As the turntable rotates, the direction of the pheromone trail diverges increasingly from the food–nest axis. If ants are to reach the nest without leaving the trail, they must at some point make a U-turn and retrace their steps on the trail and so move towards the visually defined position of the nest. Alternatively, ants could abandon the trail and aim continuously at the visually defined nest position (figures 1 and 2*a*). The rotation speed was 3 r.p.m. (EXP 1-1). This rotational speed meant that ant was about halfway across the turntable by the time the trail had rotated through 90°. We also observed what the ants did when the turntable was stationary (figures 1 and 4*a*), which yielded data for the control experiment (EXP 1-2). The foraging lane was pheromone-coated.

#### 2.1.2 Experiment 2: low-speed rotating table

**Figure 5.**
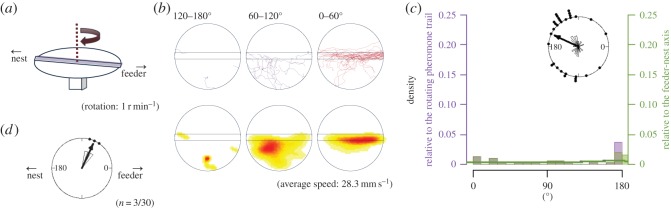
The apparatus and results from EXP 2. Explanations of diagrams and graphs (*a*–*d*) are the same as in figure 2, except different explanations are added as follows: (*a*) the rotational structure in the experimental apparatus of EXP 2. (*c*) In EXP 2, the histogram relative to the feeder–nest axis is homogeneously distributed and the peak of the histogram relative to the rotating pheromone lane is around 180° (i.e. most ants reached the opposite side of the lane).

We investigated the ants' behaviour when the speed of rotation was sufficiently slow (1 r.p.m.) for the ants to reach the nest side without making a U-turn (figures 1 and 5*a*). The aim was to determine whether the behaviours in EXP 1 were affected by the rotation. The rotation speed was 1 r.p.m., and the ants reached the other edge within a quarter of a rotation if they went straight. The foraging lane was pheromone-coated.

#### 2.1.3 Experiment 3: rotating table surrounded by a white wall

**Figure 6.**
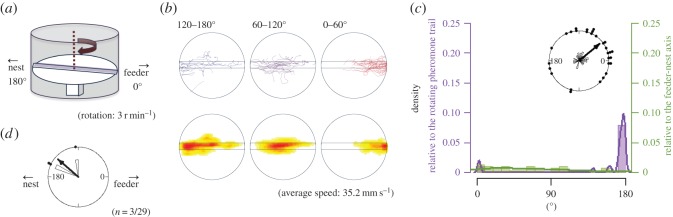
The apparatus and results from EXP 3. Explanations of diagrams and graphs (*a*–*d*) are the same as in figure 2, except different explanations are added as follows: (*a*) the rotational structure in the experimental apparatus of EXP 3. A turntable was surrounded by a white wall. (*c*) In EXP 3, the peak of the histogram of angles relative to the rotating pheromone lane is at 180° because all ants went straight to the nest side.

We surrounded the rotating table with a white paper to prevent the ants from using a panoramic perspective for guidance (figures 1 and 6*a*). The ants could enter or leave the foraging lane through a small entrance that opened in the wall. The rotation speed was 3 r.p.m., and the ants reached an edge within half a rotation if they went straight. The foraging lane was pheromone-coated.

#### 2.1.4 Experiment 4: rotating bridge

**Figure 7.**
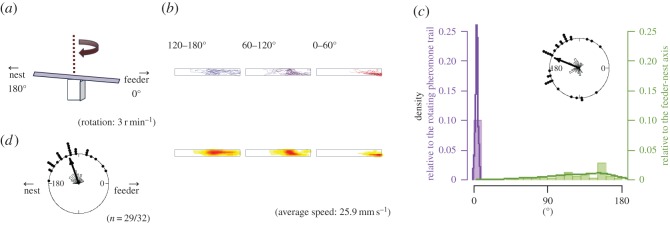
The apparatus and results from EXP 4-1. Explanations of diagrams and graphs (*a*–*d*) are the same as in figure 2, except different explanations are added as follows: (*a*) the rotational structure in the experimental apparatus of EXP 4-1. (*c*) The peak of the histogram relative to the feeder–nest axis is around 180° and that of the pheromone lane is around 0°. That indicates that most ants completed a U-turn (returned at 180° relative to the feeder–nest axis) and returned to the starting point (0° relative to the rotating pheromone lane).

**Figure 8.**
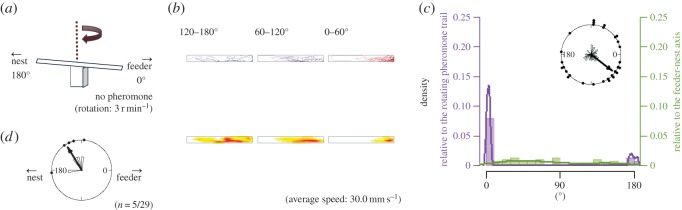
The apparatus and results from EXP 4-2. Explanations of diagrams and graphs (*a*–*d*) are the same as in figure 2, except different explanations are added as follows: (*a*) the rotational structure in the experimental apparatus of EXP 4-2.

To test the ants' behaviour when there was no pheromone trail, we used a rotating ‘bridge’ instead of a rotating disc. The width of the bridge (3 cm) was the same as the lane. In one experiment, the bridge was coated with pheromone (EXP 4-1) as shown in figures 1 and 7*a*, and in a second experiment the bridge was not treated (EXP 4-2) as shown in figures 1 and 8*a*. Rotational speed was 3 r.p.m., as in EXP 1-1.

### 2.2 Method of analysis

**Figure 9.**
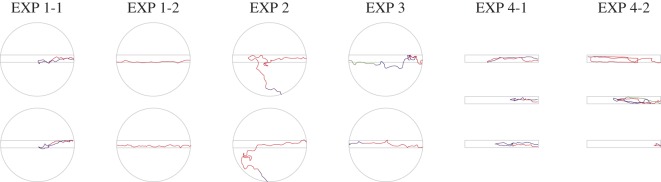
Typical trajectories. EXP 1-1 and EXP 4-1, 24/30 ants and 29/32 ants completed a U-turn. EXP 1-2 and EXP 3, 30/30 ants and 16/29 ants went straight. EXP 2, ants tended to leave the pheromone lane and succeeded in reaching the nest side. EXP 4-2, 13/29 ants drifted widely and 9/29 ants drifted narrowly.

All the foraging trajectories of one treatment were collected and their density along the trail is shown with a colour code going from red (dense) through yellow to white (least dense). In figures 2, 4–9, trajectories are given relative to the rotating disc, so that the right-hand side of the circle represents the region near the feeder end of the foraging lane. The distribution was determined using a Gaussian kernel density estimator, and a bandwidth was selected using the method from Sheather & Jones [24]. Circularly distributed data were analysed using circular statistics, based on a circular normal distribution. We defined the endpoint of an ant's trajectory on the turntable by two angles: first, the angle between the line from the endpoint to the centre of the turntable and the nest-feeder axis (figure 3*a*), and second, the angle between this line and the pheromone lane (figure 3*b*). We also noted the direction of a foraging lane when an ant made a U-turn after travelling at least one-quarter of the lane. The angle *ϕ* denotes the mean of the distribution of these three kinds of angles for an experiment and *r* is the scatter of the distribution. Watson–Williams and Rayleigh analyses were used to determine the mean homogeneity and uniformity, respectively. A binominal analysis was used to determine the deviation of the distribution of the U-turn among some experiments, and a Fisher's exact analysis was used for the non-random association between two categorical variables, the experimental condition type and the number of U-turns (both analyses were two-tailed). We used an alpha level of 0.05. To compare the shape of trajectories, we calculated the sinuosity, which is given by the path length over the lane-axial length (figure 3*c*).

## 3. Results

To compare the results, we present four components in each figure: (i) experimental set-up, (ii) arrangement of the trajectories on the lane and/or the turntable, (iii) density distribution against the angle relative to the feeder–nest axis and against the angle relative to the rotating pheromone lane, and (iv) the turning point angle distribution for each experiment (figures 2, 4–8).

### 3.1 Experiment 1: rotating table

**Figure 10.**
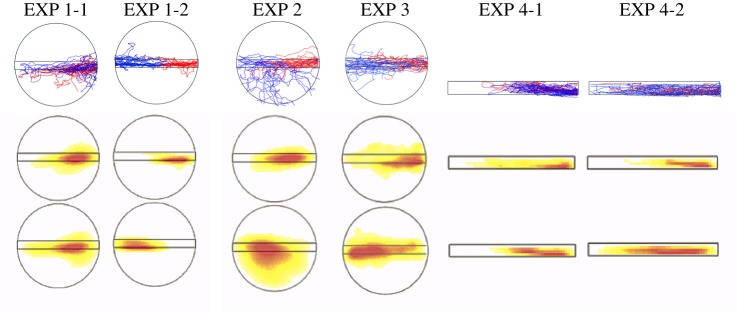
Trajectories and distribution of ants' walking in each experimental condition. Each trajectory is divided into two parts (by the middle point with respect to time). The first half of each trajectory is shown in red, and the second half is in blue (top); the density distribution of the first half (centre) and second half (bottom) of the trajectory is graded from more dense (red) to less dense (yellow to white).

Figure 2*b* shows the ants' trajectories on the lane that rotated (EXP 1-1). Figure 4*b* shows the same for EXP 1-2 when the turntable was kept stationary. Each trajectory of the first 60° rotation is shown in red, the second 60° rotation is in violet and the third 60° rotation is in blue (figure 2*b*, top row). On a rotating disc, the ants first ran along the pheromone lane towards the centre of the disc and then made a U-turn so that, relative to the rotating disc, they returned to the starting point. But relative to the stationary room, their U-shaped path brought the ants to the region of the nest. The density distributions of the ants' positions during each third of the raw trajectories are plotted in the bottom row of figure 2*b* and make the same point, as does figure 10, in which the trajectories are divided into two halves according to their duration. In contrast, the corresponding plots of the ants' paths when the table was stationary (figure 4*b* and figure 10) show that the ants walked from the feeder end to the nest end of the pheromone lane with no U-turns.

**Figure 11.**
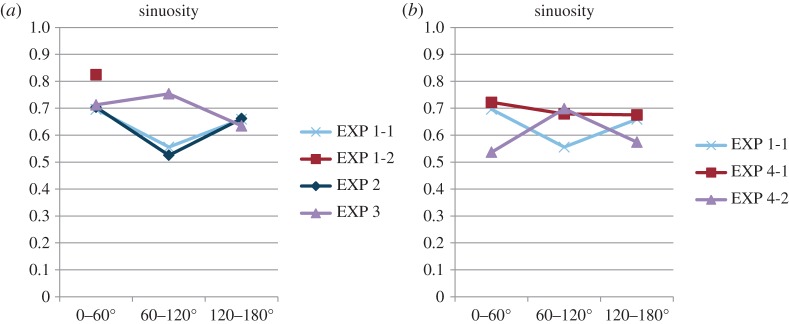
Sinuosity. For each trajectory, sinuosity is calculated as the path length over the lane-axial length of each 60° rotation (figure 3*c*), where the path length is the whole length of the trajectory, and the lane-axial length is the distance between the tangent line to turntable perpendicular to the lane-axial and the farthest point of the trajectory from it. The graph shows the mean values among each experiment. When ants completed a U-turn, we could measure low sinuosity. In EXP 1-2, most ants made straight trajectories, thus the average sinuosity was the highest. Meanwhile, in EXP 1-1 and EXP 4-1, as most ants completed a U-turn in the second portion (60–120° rotation), the sinuosity of that portion was less than that of the other portions. In EXP 2, as few ants completed a keen U-turn but most ants changed direction moderately, the sinuosity of the second portion was also low. In EXP 3, where straight trajectories and winding ones were mixed, the sinuosity of the second portion was not lower than that of the other portions, and in EXP 4-2, where most ants made winding trajectories, the sinuosity of the second portion was also not lower than that of the other portions.

The rose diagram in figure 2*c* shows that the endpoint angles relative to the feeder–nest axis are distributed on the nest side and are not randomly distributed (EXP 1-1, *φ*=169°, *r*=0.661, *p*<0.0001; EXP 1-2, *φ*=176°, *r*=0.999, *p*<0.0001). In addition, the histogram in figure 2*c* shows that in EXP 1-1, the endpoint angles relative to the foraging lane are distributed around 0°, whereas figure 4*c* shows that in EXP 1-2 they are distributed around 180°. They were not randomly distributed (EXP 1-1, *φ*=10.4°, *r*=0.661, *p*<0.0001; EXP 1-2, *φ*=176°, *r*=0.999, *p*<0.0001), and they differed significantly (*p*<0.0001). We also compared the number of U-turns with that of non-U-turns. The rotational condition and number of U-turns were significantly associated (*p*<0.0001). Thus, the ants completed U-turns more often on the rotating table than on the stationary table. In figure 2*d*, the occurrence of U-turns is plotted relative to the direction of the pheromone lane. This rose diagram indicates that ants tended to make U-turns when the pheromone lane was perpendicular to the feeder–nest direction (*φ*=98.7°, *r*=0.867, *p*<0.0001) (figure 2*d*). This result indicates that each ant changed direction when the lane was rotated approximately 90°. Moreover, in EXP 1-1, the sinuosity of the second portion (from 60° to 120° rotation) was lower than the first and third portions (figure 11). These results show that the ants changed their direction depending on the panoramic views, whereas they tended not to leave the pheromone lane (electronic supplementary material, Movies S1 and S2), and that the ants used the pheromone trail as a cue for foraging and also when using the visual cues.

### 3.2 Experiment 2: low-speed rotating table

What happens if the pheromone lane rotates so slowly (1 r.p.m.) that the ants can reach the nest region without making a U-turn? In EXP 2 (figure 5*a*), the trajectory of the first 60° rotation was distributed in various ways (figure 5*b*). The rose diagram in figure 5*c* shows that the endpoint angles relative to the panorama are broadly distributed towards the nest side (*φ*=155°, *r*=0.408, *p*=0.00579), while the endpoint angles of the pheromone lane are very roughly perpendicular to the feeder–nest axis (*φ*=118°, *r*=0.426, *p*<0.0036), differing significantly from the distributions of EXPs 1-1 and 1-2. Very few U-turns were made (figure 5*d*) and all three occurred when the pheromone lane had rotated through more than 90°. It can be seen in figure 5*b* that some ants left the pheromone trail and approached the nest (figure 9). These results show that ants can respond to visual cues and ignore the pheromone trail when the panorama moves slowly (electronic supplementary material, Movie S3).

### 3.3 Experiment 3: rotating table surrounded by a white wall

In EXP 3, the turntable rotated at 3 r.p.m. within a uniform white drum (figure 6*a*), so that the rotation would not generate any visual input. The trajectories of each 60° rotation are shown in figure 6*b*, and the first and the second half of the trajectories are shown in figure 10 (EXP 3). Both figures show that the ants gradually moved across the whole table following the pheromone trail. The rose diagram in figure 6*c* shows that the endpoint angles relative to the panorama are distributed broadly on the feeder side (*φ*=158°, *r*=0.420, *p*=0.00507). The histogram in figure 6*c* shows that the endpoint angles relative to the pheromone trail are distributed tightly on the nest side (*φ*=172°, *r*=0.786, *p*<0.0001), and they significantly differed from EXP 1-1 (*p*<0.0001). The number of U-turns was fewer than the non-U-turns (*p*<0.0001). U-turns were infrequent (in nine out of 29 paths) suggesting that U-turns do not occur without a visual scene that rotates relative to the pheromone trail (electronic supplementary material, Movie S4). Although the results of EXP 3 are similar to the results of EXP 1-2 with respect to the absence of a U-turn (figure 10), the sequential patterns of sinuosity are different from each other (figure 11*a*). This suggests that the ants tried to go straight but were unable to, causing them to wander because their ‘ground’ was rotated and shaken.

### 3.4 Experiment 4: rotating bridge

In EXP 4-1 and 4-2, the turntable was replaced by the rotating bridge (figures 7*a* and 8*a*). When the rotating bridge was coated with pheromone, the ants made a U-turn (figure 7*b*) as in figure 3*b*. Thus, they returned to the start of the pheromone trail (figure 7*b*, left) and ended up on the nest side (figure 7*c*). When the bridge was uncoated, the ants rarely made U-turns (figure 8*b*). All trajectories of each 60° in EXP 4-1 were in the right-hand portion (figure 7*b*), whereas in EXP 4-2 the trajectories were distributed in various ways (figure 8*b*, left). The similar tendency was also found in figure 10. Both the first and second half of the trajectories were distributed on the right-hand portion in EXP 4-1, which shows that the ants walked towards the nest by completing a U-turn, and the second half was distributed on the left-hand portion in EXP 4-2, which shows that some ants did not complete a U-turn. The rose diagrams in figure 7*c* show that the angles for the EXP 4-1 endpoints relative to the feeder–nest axis are distributed on the nest side and are not randomly distributed (figure 7*c*; *φ*=158°, *r*=0.530, *p*=0.0001); the EXP 4-2 endpoints are randomly distributed (figure 8*c*; *φ*=225°, *r*=0.233, *p*=0.208, n.s.). The endpoint angle distribution in EXP 4-1 differed significantly from EXP 4-2 (figure 7*c*; *F*_1,59_=38.6, *p*<0.0001), but not from EXP 1-1 (*p*=0.135, n.s.). The association between the pheromone condition and the number of U-turns was statistically significant (*p*<0.0001). Thus, ants complete more U-turns on the pheromone trail than on the non-pheromone trail. Additionally, the histograms in figures 7*c* and 8*c* show that for both EXP 4-1 and EXP 4-2, the endpoint angles relative to the rotating pheromone lane are distributed around 0° and are not randomly distributed (EXP 4-1, *φ*=4.11°, *r*=0.999, *p*<0.0001; EXP 4-2, *φ*=4.74°, *r*=0.587, *p*<0.0001).

The rose diagram in figure 7*d* shows that the turning point angles in EXP 4-1 are distributed at approximately 90° and are not randomly distributed (*φ*=109°, *r*=0.823, *p*<0.0001). This result indicates that each ant changed direction when the lane was rotated approximately 90°. Moreover, in EXP 4-1 the sinuosity of the second portion (from 60° to 120° rotation) was lower than the first and third portions, but not in EXP 4-2 (figure 11*b*). These results show that the ants required a pheromone trail during turning to follow the rotating visual panorama (electronic supplementary material, Movies S1 and S2) and suggest that ants used the pheromone trail as the primary cue and the visual information as the secondary cue in foraging.

## 4. Discussion

Garden ants, *L. niger*, live in a habitat that includes widespread visual cues, such as bushes, trees or distant cliffs; they learn quickly and use local information for guiding foraging routes [25]. Even with the complexity of maze-like trails, ants follow foraging routes and successfully return to their nest [25,26]. Our experiments included several visual cues for ant navigation, such as office furniture and experimental equipment. Ants are known to use information from the visual panorama [27–30].

Ants can navigate even with a plain white wall or curtain [31], an imitation of the skyline [32], or the global shape of the arena [33]. Because the experiments were performed on ants on their first foraging trip on the turntable, there was no interactive learning effect [34] on the foraging. Therefore, there was no motor input (vibrations or centrifugal force) effect in navigation [35] that could be estimated by comparing the results of EXP 1-2 and EXP 3. With the pheromone trail and stationary panorama view ants tended to go straight, whether they were given motor input (EXP 3) or not (EXP 1-2). Our results also suggests that ants are able to react to this rotational optic flow so as to be enable to match the visual panorama whether it rotates (EXP 1-1) or not (EXP 1-2). Rotational optic flow may be used to help the ant compensate for the rotation and maintain a straight course in regards to the panorama, as an optomotor response.

Since the pheromone trail has no polarity, in EXP1-1 the ants could get the conflict between the pheromone trail and the visual cues, when they use prior knowledge on homing direction and present visual cues. At the beginning of the trials, the ants followed the pheromone trail and walked straight because the prior visual cues (i.e. visual cues when the turntable stopped) showed the nest in front of the ant. Our experiments show that the ants followed the pheromone trail with the help of visual cues. The ants did not leave the pheromone lane whether the turntable rotated at 3 r.p.m. or not; therefore, it is clear that the ants followed the pheromone trail (figures 2, 4 and 6).

If the lane rotated very slowly at 1 r.p.m., some ants left the pheromone trail and directly approached the nest independent of the pheromone trail (figures 5 and 9). Regarding the endpoint angles relative to the rotating pheromone trail, the numbers of ants that finished their trajectory in the pheromone trail were 21 out of 30 paths in EXP 1-1, and 14 out of 30 paths in EXP 2. This result shows that in EXP 2 many more ants left the pheromone trail than in EXP 1. Therefore, there could be a threshold value for the angle between the lane and the feeder–nest axis, below which the ant can identify the conflict between the prior knowledge and the visual cues. In addition, the threshold might be dependent on the rate of changing visual cues, which was controlled by the rotation velocity of the turntable in our experiment. Thus, the ants could not identify the conflict until the angle between the lane and the feeder–nest axis reached a particular threshold of 90° (maximum) when the turntable rotated at 3 r.p.m. In contrast, the ants could identify the conflict even if the angle was very small and directly approached the nest when the turntable rotated very slowly at 1 r.p.m.

The pheromone lane was set in the turntable, therefore the ants could choose to walk either on the pheromone trail or not. We estimated whether the choice of the pheromone tail might affect the use of visual cues or not (EXP 4-1, figure 7). The results show that there was no effect. Even when the pheromone bridge was rotated at 3 r.p.m., the ants completed a U-turn at 90°. We also estimated whether changing visual cues seen from the foraging bridge could affect the ants' walking without the pheromone trail (EXP 4-2, figure 8). In this experiment, few ants completed U-turns, i.e. without odour, the ants did not complete a U-turn and appeared confused by the rotation. This result indicates that a pheromone trail provides a scaffold to the ants for efficient and stable completion of complex tasks and is consistent with studies that suggest a synergy between pheromone trails and route memory [25]. This synergy increases foraging efficiency in ants [15]. It suggests that the pheromone trail could yield a necessary condition to perceive changing visual cues.

One can roughly estimate the presence or absence of a U-turn and/or foraging pattern on the lane by comparing those factors for all experimental conditions (figure 10). EXP 1-1 and 4-1 show that both the first and the second half of the ants' walk are located at the right-hand part of the lane. This implies that in figure 10, ants walked from the feeder end of the lane towards the centre of the lane in the first half and from the centre to back to the feeder end of the lane which had now rotated to face the nest. In control experiments, when the turntable was stationary or moved slowly relative to the visual surroundings, ants tended to walk from the feeder end of the pheromone lane all the way to the other end. EXP 1-2, 2 and 3 in figure 10 show that the walking pattern shifted from the right part to the left part of the lane, which implies that an ant walks straight from the right end to the left end. EXP 4-2 in figure 10 shows that ants' walking patterns are so diverse that the walking pattern is homogeneously distributed over the lane. Figure 9 shows typical walking patterns for each experimental condition. Each condition contains two patterns. The trends obtained in figure 10 are also found in each walking pattern. It is easy to see that an ant left the lane and directly approached the nest in EXP 2 (i.e. slow rotation).

With respect to sinuosity in each 60° phase, graphs of EXP 1-1 and 2 show the same tendency and that of EXP 3 is different to the former ones (figure 11*a*). This result is reasonable because sinuosity decreased in ants that completed a U-turn in the middle phase of EXP 1-1 and 2, whereas EXP 3 did not show a decrease due to no U-turn. Figure 11*b* shows a comparison between EXP 1-1 and both EXP 4-1 and EXP 4-2 with respect to sinuosity. Sinuosity in EXP 4 showed no decrease in the middle phase. This is also reasonable because of a very fast U-turn in EXP 4-1 and no U-turn in EXP 4-2.

We investigated the paths taken by ants homing on a rotating table or lane. We constructed a novel experimental design to provide previous visual information guided by an olfactory cue and a present visual cue in conflict in a continuous manner, we performed appropriate control conditions, and, crucially, we performed this without interfering directly with the animal. Our results show that ants can modify their homing behaviour dependent on a changing visual cue and that sensitivity to a changing visual cue is affected by the olfactory cue and changing rate. These results should contribute greatly to our understanding of how information from different modalities is integrated in ants.

## Supplementary Material

Fig_ESM1

## Supplementary Material

Fig_ESM2
