# Supplementary figures and images for: Rotating panoramic view: interaction between visual and olfactory cues in ants
Source: R Soc Open Sci. 2016 Jan 27;3(1):150426. doi: 10.1098/rsos.150426 (PMC4736924; doi:10.1098/rsos.150426)

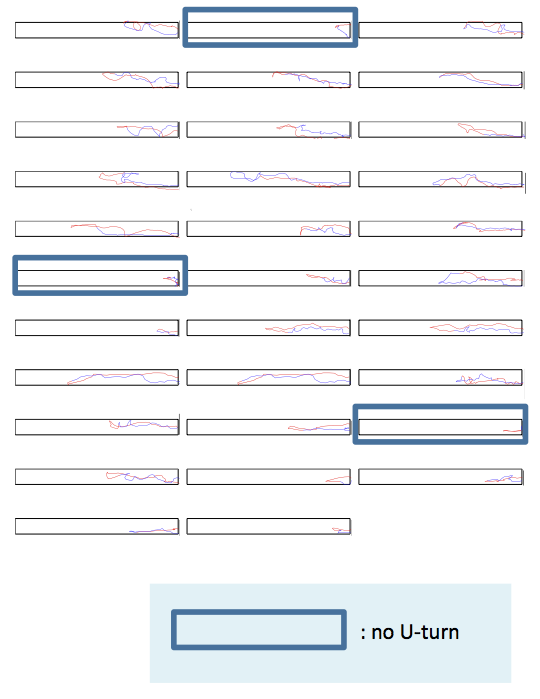

Supplement: Fig_ESM1 [file rsos150426supp1.tiff]

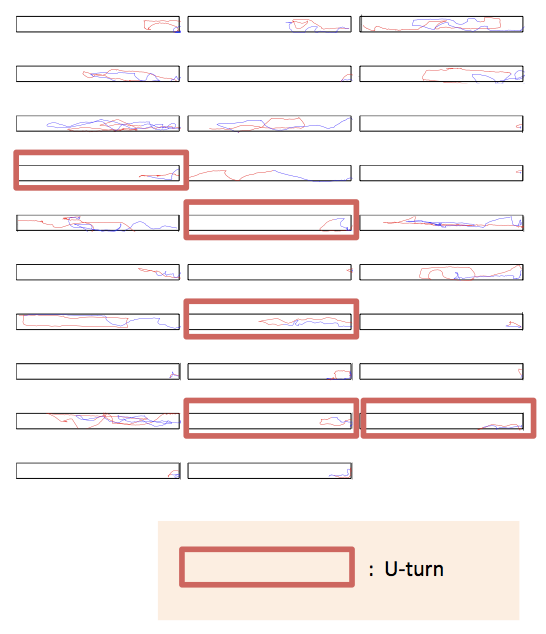

Supplement: Fig_ESM2 [file rsos150426supp2.tiff]
